# Daily mother-infant skin-to-skin contact and maternal mental health and postpartum healing: a randomized controlled trial

**DOI:** 10.1038/s41598-022-14148-3

**Published:** 2022-06-17

**Authors:** Kelly H. M. Cooijmans, Roseriet Beijers, Bonnie E. Brett, Carolina de Weerth

**Affiliations:** 1 Department of Developmental Psychology, Behavioural Science Institute, Radboud University, Thomas van Aquinostraat 4, 6525 GD Nijmegen, The Netherlands; 2 Department of Cognitive Neuroscience, Donders Institute for Brain, Cognition, and Behavior, Radboud University Medical Center, Nijmegen, The Netherlands

**Keywords:** Psychology, Health care

## Abstract

This randomized controlled trial examined the effects of a daily hour of mother-infant skin-to-skin contact (SSC) during the first five postnatal weeks, compared to care-as-usual, on maternal depressive (primary outcome), anxiety, stress, fatigue, pain, and delivery-related post-traumatic stress symptoms (PTSS). Prenatal symptom severity and touch discomfort were examined as moderators. Mothers and full-term infants were randomly allocated to SSC or care-as-usual conditions and followed during the first postnatal year. For the total group (intention-to-treat analyses), care-as-usual mothers showed an increase of anxiety symptoms from week 2 to 12, while SSC mothers displayed a stability of anxiety symptoms. Also, care-as-usual mothers showed an initial decrease in fatigue followed by an increase, while SSC mothers showed a decrease from week 2 to 12. In per-protocol analyses, including only the SSC dyads who adhered to SSC guidelines, findings on anxiety, but not fatigue, were replicated. No SSC effects were found for depressive, stress, and pain symptoms. No moderator, dose–response, or 52-week follow-up effects were found. PTSS were low with little variation; consequently, analyses were discontinued. Daily SSC in healthy mother-infant dyads may reduce anxiety and fatigue symptoms, but not depressive, stress, and pain symptoms, during the early postpartum period. Replication studies are recommended.

## Introduction

The period following childbirth, while joyful, can be challenging. As a consequence, the postpartum period is often accompanied by clinical and subclinical maternal mental health symptoms. Postnatally, 17–19% of all mothers experience depressive symptoms, 6–15% experience anxiety symptoms, and 5–38% experience stress symptoms^1–4^. Such symptoms are associated with suboptimal child development in the short- and long-term^5,6^. Diverse intervention programs exist to alleviate these symptoms but their effectiveness is mixed^7,8^. Moreover, even clinical levels of maternal mental health difficulties can remain undetected or untreated for a long period or may never be addressed^9^. The postnatal period is also one of healing, with many mothers experiencing delivery-related symptoms. In the first months postpartum, 40 to 60% of women report elevated fatigue and up to 72% report delivery-related pain^10–13^. Additionally, up to 30% of women experience delivery-related post-traumatic stress symptoms (PTSS) in the weeks after birth^14–16^. In turn, these postpartum symptoms are also linked with suboptimal outcomes such as cessation of breastfeeding, decreased ability to care for the infant, and a negative perception of the infant^10–13,17^. Accordingly, it is critical to discover inexpensive and accessible avenues to aid women in managing the postpartum period.

Daily skin-to-skin contact (SSC), placing the diapered infant onto the mother’s bare chest^18^, has the potential to decrease postnatal maternal mental health difficulties and improve maternal postpartum healing. The SSC method was originally developed for preterm and low birthweight infants as an alternative for incubator care and found to be associated with even better infant outcomes, including infant health and regulation^19,20^. Daily SSC in preterm infants also has positive effects on the mother, such as improved bonding and increasing initiation and duration of breastfeeding^20,21^. Moreover, two studies in preterm infants associated daily SSC with lower levels of maternal depressive symptoms; another found it linked with lower levels of anxiety and stress during the first six postnatal months^21–23^. For similar reasons, SSC is now also encouraged in healthy full-term infants during the first postnatal hours and days^24^.

In mothers of full-term infants, only two studies examined SSC effects on maternal mental health symptoms. In one, SSC immediately after birth was related to fewer maternal anxiety symptoms three days postpartum^25^. The other examined the effectiveness of a long-term repeated SSC intervention in a healthy subclinical sample^26^. SSC mothers, compared to control mothers, reported significantly fewer depressive symptoms at one week and marginally fewer at one month postpartum. No differences were found at two and three months postpartum.

Less is known on effects of SSC on maternal postpartum healing. In healthy infants, SSC immediately after birth was associated with decreased pain responses and improved physiological health indices for the infant^25,27^. SSC effects on maternal healing focus only on the immediate effects of one-time SSC following delivery: reduced bleeding, earlier placental expulsion, increased energy, and a shorter hospital stay^28–31^. These results are promising, but these studies did not use a randomized controlled design, nor did they examine the effects of daily, repeated SSC.

Though the working mechanisms that relate SSC to improved maternal mental health and postpartum healing are mainly unknown, SSC activates nerve fibers through touch; in turn, this activates the oxytocinergic system through which the hormone oxytocin is released in the maternal body^32–34^. Elevated oxytocin concentrations may be able to decrease HPA-axis and sympathetic nervous system activity, hence reducing physiological stress reactions^32,34^. Higher oxytocin levels are also related to improved maternal mental health, including reduced stress and anxiety symptoms^32^. Additionally, given that prolonged exogenous administration of oxytocin has been shown to alleviate post-traumatic stress disorder symptoms and has been suggested as a potential treatment for delivery-related PTSS, daily SSC may decrease delivery-related PTSS^35,36^. In addition to the activation of the oxytocinergic system, during SSC mothers also experience a period of rest which may facilitate maternal healing. Finally, prior research demonstrated that interventions involving touch and relaxation, both integral to SSC, have the potential to reduce pain and improve sleep, physical health, and wound healing following surgery in healthy adults^38–41^.

The current randomized controlled trial (RCT; protocol publication^42^) investigated whether a five-week daily hour of SSC, compared to care-as-usual (CAU), between mothers and their healthy, full-term infants influenced maternal depressive (primary outcome), anxiety, stress, fatigue, pain, and delivery-related PTSS symptoms at 2, 5, and 12-weeks postpartum and at a 52-week follow-up. Further, prenatal depressive, anxiety, and stress complaints were included as potential moderators in the analyses as they may have specific effects on the efficacy of the intervention. While psychological treatments for depression may be more efficacious with high pretreatment severity compared to low pretreatment severity [see meta-analysis;^43^], higher pretreatment anxiety symptoms seem to predict a poorer treatment outcome [see review;^44^]. Finally, given that individuals vary on their discomfort associated with touch^45^, which may affect intervention effectiveness, touch discomfort was examined as a second potential moderator for all outcomes. We hypothesized that: (1) daily SSC, compared to CAU, would reduce maternal postnatal mental health symptoms and improve maternal postnatal healing; (2) in SSC mothers, higher prenatal depressive symptoms would predict lower post-intervention depressive symptoms, while higher prenatal anxiety symptoms would predict higher post-intervention anxiety symptoms; (3) Higher levels of touch discomfort would predict higher levels of postnatal mental health symptoms and poorer healing outcomes in SSC mothers. Moderator effects of prenatal stress symptoms were exploratorily examined. Since no research is available about long-term effects of SSC on maternal mental health and postpartum healing, no hypotheses are advanced regarding effects at 52-weeks postpartum; these analyses are viewed as exploratory.

## Results

### Enrollment

**Figure 1:**
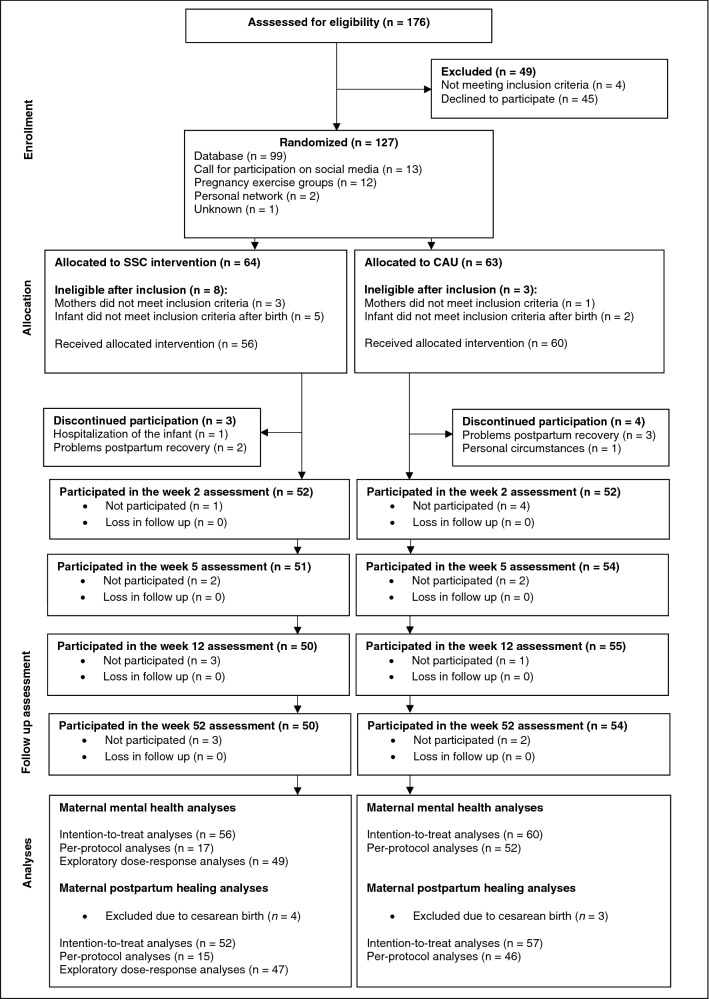
Flow diagram, including participant numbers through each stage of the trial. Numbers represent mothers that participated in the measurement round but do not define the number of mothers that actually filled out the maternal mental health and postpartum healing questionnaires. SSC, skin-to-skin contact; CAU, care-as-usual.

Between April 2016 and September 2017, 127 women were enrolled during late pregnancy (n_SSC_ = 64; n_CAU_ = 63). Initially, only 116 women were recruited. After enrollment, four women were excluded due to prenatal medication use and seven infants did not meet the eligibility criteria after birth. Consequently, eleven additional pregnant women were recruited. During the intervention period, seven mothers discontinued participation, but no loss to follow-up was observed. The 52-week follow-up was between May 2017 and October 2018. At the 52-week follow-up, 95% of the SSC and 93% of the CAU mothers were still part of the study. For the fatigue, pain, and delivery-related PTSS analyses, mothers delivering via caesarian section (n = 7) were excluded because the number of mothers who had a cesarean was small and the cesarean surgical procedure often involves a different and longer recovery period compared to vaginal births. As a result, 116 dyads were included in the maternal mental health analyses (n_SSC_ = 56; n_CAU_ = 60) and 109 dyads were included in the fatigue, pain, and delivery-related analyses (n_SSC_ = 52; n_CAU_ = 57). Mothers reported no adverse events. The participant flow is presented in Fig. 1.

### Preliminary analyses

Descriptive statistics for all study participants (including those who delivered via caesarian) and sample baseline characteristics are presented in Table 1. Inspection of the data set delivered a total of 34 outliers out of 4525 datapoints; these data points were winsorized^46^. Across all variables, 7% of the data were missing. Missingness was not completely at random (Little’s MCAR test: *χ*^2^ = 612.30, *df* = 476, *p* = 0.001). Missing data were handled using maximum likelihood estimation (the default for multilevel models). Detailed information on the missingness and outliers is presented in Supplementary Table S1 online.

**Table 1:** Baseline characteristics and potential moderators of randomized participants.

	Intention-To-Treat		Per-Protocol	
	*CAU (n* = *60)*	*SSC (n* = *56)*	*CAU (n* = *50)*	*SSC (n* = *17)*
**Baseline characteristics**				
Maternal age, mean ± SD years	32.48 ± 3.05	32.36 ± 3.85	32.50 ± 3.22	32.96 ± 3.91
Maternal educational level, mean ± SD	6.87 ± 1.79	6.82 ± 1.55	7.02 ± 1.61	6.76 ± 1.52
Smoking, *n* (%)				
Yes	0(0)	2(4)	0(0)	1(6)
No	60(100)	54(96)	50(100)	16(94)
Alcohol, *n* (%)				
Yes	0(0)	1(2)	0(0)	1(6)
No	60(100)	55(98)	50(100)	16(94)
C-section, *n* (%)				
Yes	3(5)	4(7)	2(4)	1(6)
No	55(92)	51(91)	48(96)	16(94)
Missing	2(3)	1(2)	0(0)	0(0)
Birth order, *n* (%)				
First	28(47)	27(48)	20(40)	6(35)
Second	23(38)	18(32)	22(44)	6(35)
Third	9(15)	11(20)	8(16)	5(29)
APGAR score	9.70 ± 0.62	9.84 ± 0.42	9.76 ± 0.52	9.71 ± 0.59
Child sex, *n* (%)				
Male	34(57)	23(41)	27(54)	7(41)
Female	26(43)	33(59)	23(46)	10(59)
Gestational age at birth, mean ± SD, weeks	40.02 ± 1.10	40.08 ± 1.01	39.96 ± 1.08	40.15 ± 1.06
Birthweight, mean ± SD, gram	3,567.47 ± 385.77	3,650.05 ± 414.93	3,577.12 ± 361.39	3,758.24 ± 468.47
**SSC**				
Total duration SSC, mean ± SD, minutes	308.17 ± 442.41	2,067.68 ± 850.65	308.17 ± 442.41	2,844.78 ± 437.66
**Potential moderators**^**a**^				
*Maternal Prenatal (week34–36)*				
Depressive symptoms, mean ± SD ^b^	4.68 ± 3.89	4.88 ± 3.77	4.46 ± 3.64	4.12 ± 2.74
Anxiety symptoms, mean ± SD ^c^	29.25 ± 6.09	30.49 ± 7.21	28.80 ± 5.35	29.44 ± 6.74
Stress symptoms, mean ± SD	14.34 ± 7.80	15.23 ± 8.29	13.96 ± 7.25	16.12 ± 7.70
Touch discomfort, mean ± SD	29.07 ± 8.98	27.84 ± 8.44	28.93 ± 9.13	30.12 ± 8.12

*CAU* care-as-usual, *SSC* skin-to-skin contact.

^a^Winsorized data is presented for all moderator variables that included outliers.

^b^A threshold of 10 indicates clinical levels of depression.

^c^A threshold of 40 indicates clinical levels of anxiety.

Mann–Whitney U tests revealed that SSC mothers provided significantly more minutes of SSC to their infant than CAU mothers during the intervention period in both the ITT and PP analyses (*U*_*ITT*_ = 78.00*, P*_*ITT*_ < 0.001; *U*_*PP*_ = 0.00, *P*_*PP*_ < 0.001). Only 17 out of 56 SSC dyads reported ≥ 28 out of the total of 35 days (i.e. 5-week-period) with ≥ 60 SSC minutes and had no missing outcome data. A less stringent criteria of 45 min of SSC per day resulted in only four additional SSC mothers in the PP analyses. Of the total sample, 47 SSC mothers provided at least 9 days with ≥ 60 min of SSC during the intervention period, whereas only two CAU mothers did so. Baseline characteristics were not significantly different between SSC mothers who did and did not adhere to the SSC protocol.

**Figure 2:**
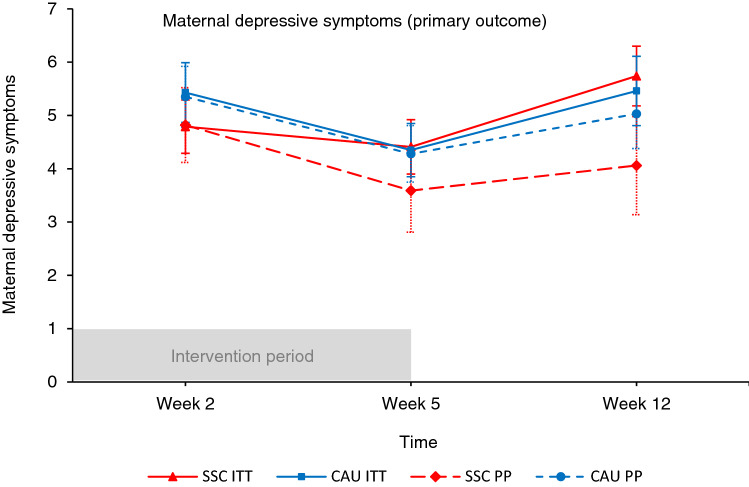
Maternal postnatal depressive symptoms during the first twelve postnatal weeks in the skin-to-skin contact (SSC) and care-as-usual (CAU) condition for the intention-to-treat (ITT) selection and per-protocol (PP) selection. Winsorized data is presented for all variables that included outliers. Error bars represent standard errors of the mean. Analyses showed that the trajectory of maternal depressive symptoms over the first twelve postnatal weeks was not different between SSC mothers and CAU mothers.

Of all participants, 4–15% reported depressive symptoms above the clinical cutoff and 8–15% reported anxiety symptoms above the clinical cutoff at different assessment rounds (see Table 2). The trajectory with means and standard errors for maternal depressive symptoms over the first twelve postnatal weeks is presented in Fig. 2.

**Table 2:** Outcome measures of randomized participants.

	Intention-to-treat		Per-protocol	
	*CAU (n* = *60)*^*a*^	*SSC (n* = *56)*^*a*^	*CAU (n* = *52)*	*SSC (n* = *17)*
**Maternal Postnatal week 2 **^**b**^				
Depressive symptoms ^c^	5.43 ± 4.06	4.79 ± 3.61	5.35 ± 4.06	4.82 ± 2.90
Anxiety symptoms ^d^	28.00 ± 6.56	29.27 ± 6.78	27.78 ± 6.42	28.82 ± 5.31
Stress symptoms	12.09 ± 10.08	12.02 ± 8.66	11.32 ± 9.43	12.41 ± 6.13
Fatigue	53.94 ± 16.82	53.13 ± 12.28	53.94 ± 16.82	50.37 ± 13.11
Pain	3.83 ± 1.13	3.54 ± 1.09	3.83 ± 1.13	3.38 ± 1.15
Delivery-related PTSS	1.30 ± 0.31	1.27 ± 0.25	1.30 ± 0.31	1.29 ± 0.23
**Maternal Postnatal week 5 **^**b**^				
Depressive symptoms ^c^	4.35 ± 3.68	4.41 ± 3.66	4.28 ± 3.77	3.59 ± 3.20
Anxiety symptoms ^d^	29.78 ± 8.09	28.91 ± 7.46	29.66 ± 8.34	28.48 ± 6.48
Stress symptoms	10.34 ± 8.27	11.37 ± 8.58	10.34 ± 8.53	10.94 ± 6.87
Fatigue	50.64 ± 15.86	47.95 ± 13.27	50.64 ± 15.86	45.43 ± 13.43
Pain	2.51 ± 1.20	2.13 ± 0.96	2.51 ± 1.20	1.88 ± 0.98
Delivery-related PTSS	1.29 ± 0.34	1.22 ± 0.23	1.29 ± 0.34	1.23 ± 0.25
**Maternal Postnatal week 12 **^**b**^				
Depressive symptoms ^c^	5.46 ± 4.81	4.74 ± 3.95	5.03 ± 4.62	4.06 ± 3.80
Anxiety symptoms ^d^	30.33 ± 7.69	29.18 ± 9.01	30.12 ± 7.89	27.24 ± 5.76
Stress symptoms	11.86 ± 9.21	11.95 ± 9.90	11.57 ± 9.30	9.94 ± 6.48
Fatigue	55.48 ± 17.90	45.24 ± 13.31	55.48 ± 17.90	44.81 ± 10.84
Pain	1.97 ± 0.96	1.88 ± 1.07	1.97 ± 0.96	1.91 ± 1.30
Delivery-related PTSS	1.28 ± 0.33	1.22 ± 0.22	1.28 ± 0.33	1.21 ± 0.17
**Maternal Postnatal week 52 **^**b**^				
Depressive symptoms ^c^	4.94 ± 3.90	4.09 ± 3.94	4.67 ± 3.77	4.47 ± 3.89
Anxiety symptoms ^d^	30.31 ± 8.26	29.42 ± 8.82	29.88 ± 8.07	30.12 ± 6.30
Stress symptoms	14.75 ± 11.16	14.25 ± 10.50	14.27 ± 11.30	12.59 ± 8.97
Fatigue	53.71 ± 16.80	49.11 ± 14.63	53.71 ± 16.80	50.82 ± 16.31
Pain	2.15 ± 0.92	2.08 ± 1.09	2.15 ± 0.92	2.15 ± 1.30
Delivery-related PTSS	1.30 ± 0.35	1.21 ± 0.22	1.30 ± 0.35	1.24 ± 0.23

*CAU* care-as-usual, *PTSS* post-traumatic stress symptoms, *SSC* skin-to-skin contact.

^a^Numbers represent the total group. For fatigue, pain, and delivery-related PTSS, 52 mothers are included in the SSC group and 57 mothers in de CAU group due to the exclusion of 7 mothers who gave birth via cesarean.

^b^Winsorized data is presented for all moderator variables that included outliers.

^c^A threshold of 10 indicates clinical levels of depression.

^d^A threshold of 40 indicates clinical levels of anxiety.

### Primary analyses

The ICC was sufficient (0.14–0.74) for all models, indicating that multilevel growth curve models were appropriate^47^. In the PP analyses with anxiety symptoms and all analyses with pain as an outcome, including the random effect of time produced warnings about the accuracy of results; accordingly, time was entered as a fixed effect. Across all measurements (i.e. week 2, 5, 12, and 52), within both conditions (i.e. the SSC and control condition) and in both analyses (i.e. the intention-to-treat and per-protocol analyses), the mean score for delivery-related PTSS ranged between 1.21 and 1.30 with a standard deviation range between 0.17 and 0.35. Ranges and median values for both conditions in the intention-to-treat and per-protocol analyses are presented in Supplementary Table S2 online. As we aimed to explain variation in women’s PTSS experiences over time, but apparently almost no variation in PTSS existed, this line of inquiry was discontinued and no further analyses were performed on the PTSS data. Final models are summarized in Tables 3 and 4. Detailed information on the fit indices throughout the model building process is presented in Supplementary Tables S3 and S4 online.

**Table 3:** Best fitting multilevel models predicting maternal depressive, anxiety, and stress symptoms from week 2 to week 12 postnatally.

	Intention-to-Treat			Per-Protocol			Exploratory Dose–Response		
	*Estimate (SE)*	*p*	95% CI	*Estimate (SE)*	*p*	95% CI	*Estimate (SE)*	*p*	95% CI
**Depressive symptoms**									
*Fixed effects*									
Intercept	1.38 (1.04)	0.187	[− 0.68, 3.45]	2.95 (1.51)	0.055	[− 0.06, 5.95]	1.00 (1.35)	0.460	[− 1.69, 3.69]
Time	− 0.06 (0.18)	0.731	[− 0.42, 0.30]	− 0.18 (0.20)	0.363	[− 0.57, 0.21]	0.03 (0.30)	0.907	[− 0.56, 0.63]
Condition ^a^	0.48 (0.55)	0.387	[− 0.62, 1.57]	0.42 (0.83)	0.611	[− 1.23, 2.08]	− 0.01 (0.01) ^b^	0.274	[− 0.001, 0.001]
Touch discomfort	0.03 (0.03)	0.351	[− 0.03, 0.09]	− 0.02 (0.04)	0.632	[− 0.10, 0.06]	0.07 (0.04)	0.054	[− 0.001, 0.15]
Prenatal symptoms	0.52 (0.08)	0.001	[0.37, 0.67]	0.55 (0.11) ^c^	0.001	[0.34, 0.76]	0.45 (0.09)	0.001	[0.27, 0.63]
*Random effects*									
Intercept	5.77 (2.84)	0.042	[2.20, 15.12]	5.52 (1.70)	0.001	[3.01, 10.11]	6.05 (4.17)	0.147	[1.57, 23.36]
Time	0.93 (0.57)	0.105	[0.28, 3.12]	0.56 (0.30)	0.063	[0.20, 1.61]	1.79 (0.98)	0.069	[0.61, 5.26]
*Deviance*	1512.05			977.38			696.03		
**Anxiety symptoms**									
*Fixed effects*									
Intercept	11.63 (3.27)	0.001	[5.15, 18.11]	12.42 (4.64)	0.009	[3.17, 21.66]	14.30 (4.10)	0.001	[6.05, 22.55]
Time	− 0.13 (0.45)	0.767	[− 1.03, 0.76]	− 0.94 (0.68)	0.171	[− 2.28, 0.41]	0.05 (0.49)	0.914	[− 0.94, 1.05]
Condition ^a^	− 1.69 (1.52)	0.269	[− 4.72, 1.33]	− 2.07 (2.23)	0.356	[− 6.47, 2.34]	− 0.01 (0.01) ^b^	0.238	[− 0.003, 0.001]
Touch discomfort	0.12 (0.06)	0.065	[− 0.01, 0.25]	0.03 (0.08)	0.622	[− 0.12, 0.19]	0.11 (0.08)	0.188	[− 0.06, 0.28]
Prenatal symptoms	0.47 (0.09)	0.001	[0.30, 0.64]	0.54 (0.12) ^c^	0.001	[0.30, 0.78]	0.43 (0.10) ^c^	0.001	[0.23, 0.63]
Time by Condition	1.30 (0.64)	0.043	[0.04, 2.56]	2.06 (0.78)	0.010	[0.51, 3.61]			
*Random effects*									
Intercept	18.94 (9.93)	0.057	[6.77, 52.93]	25.37 (5.35)	0.001	[16.78, 38.37]	20.18 (11.66)	0.084	[6.50, 62.63]
Time	1.87 (1.84)	0.308	[0.27, 12.79]				5.46 (2.61)	0.036	[2.14, 13.94]
*Deviance*	1870.43			1197.18			843.84		
**Stress symptoms**									
*Fixed effects*									
Intercept	1.22 (2.53)	0.632	[− 3.80, 6.24]	3.76 (3.77)	0.322	[− 3.76, 11.28]	1.44 (3.21)	0.656	[− 5.00, 7.87]
Time	− 0.03 (0.37)	0.942	[− 0.75, 0.70]	− 0.28 (0.43)	0.513	[− 1.15, 0.58]	− 0.18 (0.55)	0.745	[− 1.28, 0.92]
Condition ^a^	0.25 (1.19)	0.831	[− 2.11, 2.62]	1.02 (1.79)	0.571	[− 2.56, 4.60]	− 0.01 (0.01) ^b^	0.595	[− 0.002, 0.001]
Touch discomfort	− 0.01 (0.07)	0.843	[− 0.15, 0.12]	− 0.05 (0.09)	0.551	[− 0.23, 0.12]	− 0.02 (0.09)	0.806	[− 0.20, 0.15]
Prenatal symptoms	0.71 (0.08)	0.001	[0.56, 0.86]	0.59 (0.11) ^c^	0.001	[0.38, 0.81]	0.78 (0.08) ^c^	0.001	[0.61, 0.95]
*Random effects*									
Intercept	47.59 (12.14)	0.001	[28.86, 78.47]]	63.82 (15.80)	0.001	[39.28, 103.67]	18.13 (15.10)	0.230	[3.54, 92.80]
Time	6.45 (2.11)	0.002	[3.40, 12.24]	7.39 (1.31)	0.001	[4.01, 13.63]	5.27 (3.33)	0.114	[1.53, 18.20]
*Deviance*	1908.95			1239.41			889.71		

^a^Condition: 0 = CAU; 1 = SSC.

^b^Values are for dose variable rather than condition variable.

^c^For the best fitting final multilevel models, prenatal mental health symptoms were included before touch discomfort.

**Table 4:** Best fitting multilevel models predicting maternal fatigue and pain symptoms from week 2 to week 12 postnatally.

Fatigue									
**Fixed effects**									
Intercept	48.81 (1.90)	0.001	[45.05, 52.57]	51.41 (1.84)	0.001	[47.74, 55.09]	50.14 (1.41)	0.001	[47.35, 52.95]
Time	− 0.72 (0.21)	0.001	[− 1.13, − 0.31]	0.03 (0.18)	0.866	[− .33, 0.39]	− 0.32 (0.14)	0.024	[− 0.60, − 0.04]
Condition	3.89 (2.66)	0.147	[− 1.40, 9.17]				− 0.01 (0.01) ^b^	0.471	[− 0.003, 0.002]
Touch discomfort	0.27 (0.17)	0.108	[− 0.06, 0.60]				0.39 (0.17)	0.024	[0.05, 0.73]
Time by Condition	0.94 (0.29)	0.002	[0.37, 1.52]						
**Random effects**									
Intercept	153.36 (24.57)	0.001	[112.03, 209.92]	189.23 (37.43)	0.001	[128.41, 278.85]	151.83 (25.90)	0.001	[108.68, 212.11]
Time	1.01 (0.32)	0.002	[0.55, 1.88]	0.98 (0.39)	0.012	[0.45, 2.14]	0.69 (0.29)	0.02	[0.31, 1.55]
*Deviance*	2171.75			1432.63			1900.90		

**Pain**									
*Fixed effects*									
Intercept	2.52 (0.12)	0.001	[2.29, 2.76]	2.69 (0.11)	0.001	[2.47, 2.92]	2.59 (.09)	0.001	[2.41, 2.77]
Time	− 0.15 (0.01)	0.001	[− 0.18, − 0.13]	− 0.15 (0.02)	0.001	[− 0.18, − 0.11]	− 0.15 (0.01)	0.001	[− 0.18, − 0.12]
Condition	0.24 (0.17)	0.152	[− 0.09, 0.58]				0.00 (0.00)^b^	0.672	[0.00, 0.00]
Touch discomfort	− 0.01 (0.01)	0.271	[− 0.03, 0.01]				− 0.01 (0.01)	0.453	[− 0.03, 0.01]
*Random effects*									
Intercept	0.39 (0.10)	0.001	[0.23, 0.65]	0.47 (0.14)	0.001	[0.26, 0.86]	0.40 (0.11)	0.001	[0.23, 0.68]
*Deviance*	834.90			556.94			740.51		

^a^Condition: 0 = CAU; 1 = SSC.

^b^Values are for dose variable rather than condition variable.

#### Intention-to-treat

**Figure 3:**
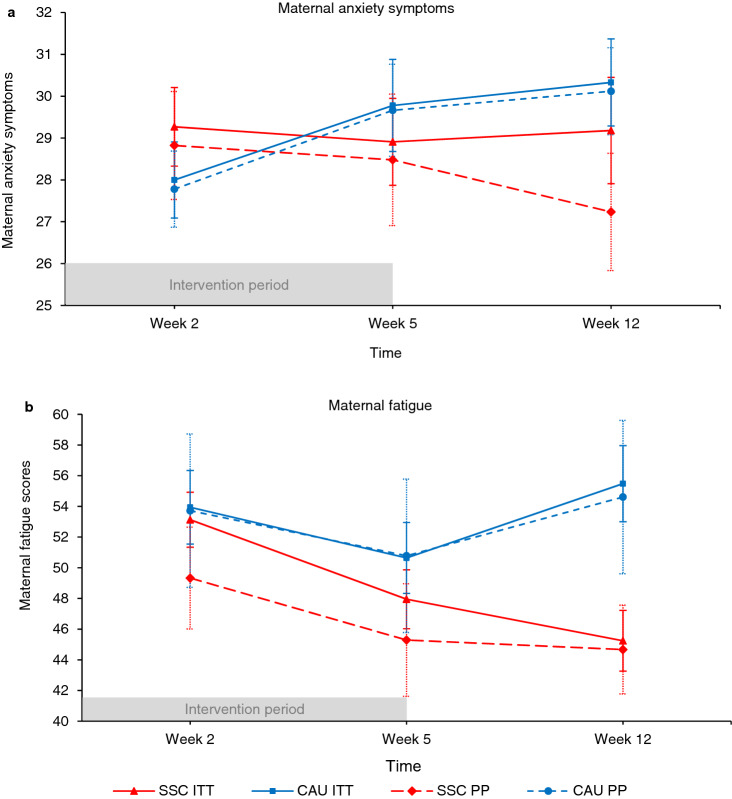
Maternal postnatal anxiety (**a**) and fatigue (**b**) symptoms during the first twelve postnatal weeks in the skin-to-skin contact (SSC) and care-as-usual (CAU) condition for the intention-to-treat (ITT) selection and per-protocol (PP) selection. Winsorized data is presented for all variables that included outliers. Error bars represent standard errors of the mean. In intention-to-treat analyses, SSC mothers displayed a stability of anxiety symptoms and a decrease of fatigue over time. Care-as-usual mothers showed an increase of anxiety symptoms over time and a decrease in fatigue symptoms from week 2 to 5 followed by an increase at week 12. In per-protocol analyses, the results for anxiety symptoms were replicated but no significant effect was found for fatigue symptoms.

For maternal anxiety symptoms, the interaction of time by condition was significant. A graph of the mean trajectories over time revealed that the SSC group showed a stable trajectory of anxiety symptoms whereas the level of anxiety symptoms in the CAU group increased between week 2 and week 12 (Fig. 3). No other main or moderator effects emerged.

For maternal fatigue symptoms, there was also a significant interaction of time by condition. A graph of the mean trajectories over time revealed that the SSC group showed a steady decrease in fatigue over the study period whereas the CAU group decreased from week 2 to week 5 but increased at week 12 (Fig. 3). No other effects were found.

Remaining models revealed no significant differences between the SSC and CAU conditions on maternal depressive, stress, or pain symptoms between week 2 and week 12. No moderator effects were revealed.

#### Per-protocol

For maternal anxiety symptoms, there was a significant interaction of time by condition. A graph of the mean trajectories over time revealed that the SSC group showed a stable trajectory whereas the CAU group increased between week 2 and week 12 (Fig. 3). No other effects emerged.

Results indicated that the trajectory of depressive, stress, fatigue, or pain symptoms between postnatal week 2 and week 12 did not significantly differ in the SSC and CAU condition. No moderator effects were revealed.

#### Exploratory dose–response analyses

SSC duration did not significantly predict the trajectory of maternal depressive, anxiety, stress, fatigue, and pain symptoms between week 2 and week 12 postnatally. Additionally, no moderator effects were found.

### Exploratory follow-up analyses

After performing exploratory hierarchical regression analyses to examine effects at 52-weeks postpartum, normality of residuals was examined. Residuals for maternal depressive and anxiety symptoms were not normally distributed. Square root and reciprocal transformations were performed on measures of depressive and anxiety symptoms, respectively, resulting in normally distributed residuals. Final models are summarized in Supplementary Tables S5 and S6 online. Results showed no significant main effects for condition or moderator effects in the ITT, PP, or DR frameworks when examining maternal depressive, anxiety, stress, fatigue and pain symptoms at 52 weeks postpartum.

## Discussion

This RCT examined the effectiveness of daily SSC for five weeks postpartum in healthy mothers of full-term infants, compared to CAU, for decreasing maternal depressive, anxiety, stress, fatigue, pain, and delivery-related PTSS symptoms during the first twelve postnatal weeks and at a 52-week follow-up. Prenatal mental health symptom severity and touch discomfort were examined as potential moderators. In this community sample, daily SSC did not decrease maternal depressive, stress, and pain symptoms during the first twelve postnatal weeks in the total SSC group (intention-to-treat analyses), or in analyses including only the group who adhered to the daily hour of SSC (per-protocol analyses). However, when comparing the total group in the ITT analyses on anxiety and fatigue symptoms, differences were found. CAU mothers showed an increase of anxiety symptoms from week 2 to 12, while SSC mothers displayed a stability of anxiety symptoms. Also, CAU mothers showed an initial decrease in fatigue followed by an increase at week 12, while SSC mothers showed a decrease of fatigue from week 2 to 12. In PP analyses, the results for anxiety symptoms, but not fatigue, were replicated. No follow-up, moderator, or dose–response effects were found for any of the outcomes. Delivery-related PTSS were not examined due to low prevalence in this sample.

Contrary to our findings regarding maternal depressive symptoms as the primary outcome, research with a comparable subclinical sample found evidence that daily SSC reduced maternal depressive symptoms one week postpartum and marginally reduced depressive symptoms at one month postpartum. Consistent with our findings, no effects were found at two and three months^26^. Importantly, this prior examination did not use a randomized controlled design, the standard for evaluating intervention effectiveness^48^. Rather, mothers were informed about the protocol before consent, potentially creating a biased sample and placebo effects if mothers anticipated beneficial outcomes^49^. Unfortunately, no baseline measures were taken to examine this explanation. It may also be that SSC duration is important. Bigelow and colleagues^26^ detected differences in depressive symptoms one week postnatally following an intensive period of six daily SSC hours. The current study chose to ask mothers to engage in one daily SSC hour to increase protocol adherence in the RCT. Possibly, only prolonged SSC during the early postnatal period has beneficial effects on maternal depressive symptoms. Additionally, since only very few mothers reached the clinical cutoff score for depressive symptoms in both studies, future research should carefully examine the roles of symptom severity and chronicity in addition to the timing and duration of SSC.

Partly consistent with the advanced hypotheses, we found differences between SSC and CAU mothers on anxiety and fatigue symptoms during the first twelve postpartum weeks when examining the total group. Additionally, the results on anxiety symptoms were also replicated for those SSC mothers that adhered to the SSC protocol. These findings are consistent with limited prior research on one-time SSC during the first hours after birth in full-term infants and state anxiety symptoms^25^ and immediate healing^28–31^ of the mother. The present study adds that daily SSC may be an effective tool for combatting postpartum anxiety and fatigue symptoms, over and above the effects of immediate post-delivery SCC, which was also performed by many mothers in the CAU group. An alternative explanation for our findings is that SSC did not influence anxiety and fatigues symptoms, but rather that symptoms were affected by perceived support. Although both intervention and control mothers were contacted weekly, it may be that the experience of being asked about SSC implementation (issues) created a feeling of support specifically in SSC mothers. A systematic review found that telephone support for women during the early postnatal period may decrease postnatal depressive symptomatology^50^. Regardless of the possible explanations raised, results should be interpreted with caution as findings on anxiety do not replicate in exploratory DR analyses and findings on fatigue were not replicated in the PP or exploratory DR frameworks. Replication is needed before translating these results to practice.

Unlike limited prior studies in preterm infants that indicated lower levels of maternal reported parenting stress six months postnatally with daily SSC^21^, we found no effect of daily SSC on maternal reports of general stress (i.e., daily hassles such as traffic jam, many social obligations) in mothers of full-term infants over the first twelve postpartum weeks. These differences in study populations and stress measures, as well as the paucity of studies on the topic, prevent us from drawing conclusions on whether SSC can effectively reduce stress in mothers. Lower levels of physiological stress, typically measured through the hormone cortisol, immediately following SSC are found in research^34^. Moreover, Bigelow and colleagues^26^ found that daily SSC in a comparable sample was linked to lower levels of maternal physiological stress. Future RCT research examining the effects of daily SSC on stress should consider examining different types of stress, including both physiological and self-reported stress levels in multiple domains (e.g., daily stress vs. parenting stress; overall cortisol production vs. cortisol reactivity).

Contrary to expectations, daily SSC did not reduce reported pain over the first twelve postpartum weeks in any analytic framework. Prior research demonstrates that mothers with vaginal deliveries experience pain immediately after birth that rapidly dissipates one week later for most mothers^51^. As only mothers who delivered vaginally were included, potential effects of SSC on pain were possibly not visible in assessments starting 14 days post-delivery. Moreover, a review provided the first indications that SSC immediately after caesarean birth may be related to lower reported pain^52^. Given that very few mothers with cesareans were included in the study overall, we decided to exclude these dyads from the analyses. Therefore, we were unable to verify if daily SSC after caesarean birth is related to reported pain in this sample. Future work should include pain assessments during the first two postnatal weeks and more women delivering with cesarean. Additionally, given the low prevalence of PTSS in the current sample, diversified samples (e.g., mothers with complicated deliveries at risk for problems related to birth healing) could help uncover whether daily SSC might prove helpful in this domain as well.

The limited beneficial effects of daily SSC on maternal mental health and postpartum healing outcomes may be due to suboptimal adherence rates in the current trial. Despite paid maternity leave and weekly contact for support and protocol adherence, only 17 out of 56 SSC mothers were able to provide one daily hour of SSC for at least 28 out of 35 intervention days and had no missing outcome data. This was surprising because, although daily SSC is not a routine part of care in the Netherlands, previous research with a much higher participant burden (i.e., 6 daily SSC hours for the first postnatal week and 2 daily hours from postnatal week 2 to 4) also found much higher adherence rates^26^. However, we note that comparable low adherence rates are often reported for behavioural change interventions, including behavioural change interventions during the postpartum period^53,54^. Moreover, participants in the current trial were not prepared for SSC when providing consent, were allocated to their condition irrespective of their motivation to perform SSC, and were not made aware of possible SSC benefits as reported in prior research. Results may have been different had higher adherence rates for more dyads been achieved, for example by more intensive SSC guidance. Although weekly support by telephone and text message/email was arranged for all mothers, more SSC guidance was potentially needed for the SSC group. Lack of help with SSC, positioning, and clothing issues are frequently noted barriers for mothers to implement SSC in preterm infants^55^. In contrast, support from family, friends, peers, and healthcare professionals, and understanding the efficacy of SSC, are commonly reported enablers of implementation^55,56^. These barriers and enablers may also be relevant for mothers of full-term infants and should be considered in future research. However, important to note, baseline characteristics for SSC mothers who adhered were not different from SSC mothers who did not adhere to the SSC protocol. Moreover, in the current trial we found SSC to be associated with other outcomes. The PP analyses revealed that SSC mothers, compared to CAU mothers, reported 1.28 months longer exclusive breastfeeding and 2.33 months longer continued breastfeeding duration^57^. Additionally, more hours of SSC were related to longer exclusive and continued breastfeeding duration in exploratory DR analyses^57^. Given current and previous findings, future studies focusing on barriers and enablers to SSC implementation in community samples are warranted.

In the current study, no follow-up effects at 52 weeks postpartum were found. Since only very few studies examined the long-term effects of SSC on maternal mental health difficulties and a small number of studies linked daily SSC with short-term maternal physical healing, it is possible that daily SSC in the postpartum period does not affect long-term mental health and healing. For mental health, only one study found SSC effects on maternal anxiety and stress symptoms during the first six months postpartum, but not 10 years after birth^21^. Another study found daily SSC in full-term infants linked to more emotional engagement and reciprocity in the dialogues between mother and child nine years later but maternal mental health was not included^58^. For physical healing, research shows postpartum healing can take up to 18 months but that physical health problems after childbirth, e.g. extreme tiredness or exhaustion, lower and upper back pain, and breast problems, decrease over time^59^. Most mothers in the current investigation reported only low levels of physical symptoms one year postpartum. Possibly, longer-term effects of daily SSC could be found in more diverse samples at risk for persistent mental or physical health complaints.

Finally, no indications were found that prenatal symptom severity and touch discomfort interfere with the effectiveness of daily SSC. For prenatal symptom severity, effects may potentially only emerge in a more heterogenous group, with more mothers suffering from serious mental health problems, as mentioned before. For touch discomfort, it is possible that the mothers who did not feel comfortable with the touch-intensive SSC intervention were overrepresented in the SSC group with low protocol adherence. However, the fact that reported general touch discomfort scores were not significantly different between mothers who did and did not adhere to the protocol speaks against this explanation.

This study has several strengths. First, it was the first to use a RCT design. Second, the trial was preregistered (Netherlands Trial Register: NTR5697; 13/03/2016) and the protocol was published^42^, improving the transparency and replicability of our findings. Third, mothers were followed for a relatively long period with more than 93 percent of mothers continuing participation one year postpartum. Limitations should also be taken into consideration. First, low SSC protocol adherence, as mentioned before, and consequently the low sample size in the PP analyses, are a limitation of the current trial. Second, the generalizability of this study may be limited as it was performed in a homogenous community sample who were highly educated. Third, although dropout rates were low, missing data was potentially more likely in mothers with mental health difficulties and physical healing problems; indeed, these were the most common reasons cited for leaving the study. Fourth, the touch discomfort questionnaire focused on attitudes and affects towards social touch in general, which can be different from touch discomfort within the mother-infant interaction. However, prior research showed that infant physiological reactions to touch were related to parental attitudes towards touch as measured with the same questionnaire as the current study^60^. These findings suggest that parental attitudes with respect to general social touch discomfort may be a reliable predictor of parental touch within parent-infant interactions. Finally, touch discomfort was measured after the intervention period whereas ideally, it should have been measured beforehand. Importantly, however, this study provides a baseline RCT for future comparisons with other populations.

## Conclusions

To our knowledge this is the first RCT examining the effectiveness of daily SSC on short- and long-term maternal mental health and physical healing in a healthy community sample of mothers of full-term infants. We found that daily SSC, beyond CAU, does not reduce maternal depressive, stress, and pain symptoms, but may be a promising intervention to prevent an increase in anxiety symptoms and reduce fatigue symptoms over the first twelve postpartum weeks. A more heterogenous sample (i.e. at risk for mental health and postpartum healing problems; mothers who experienced cesarean birth) combined with researchers’ efforts to motivate and encourage women to perform regular SSC (e.g. by making mothers aware of the potential benefits of SSC while avoiding selection bias), may elucidate stronger and additional effects. Replication studies will be crucial to verify the current results and to uncover the full range of potential SSC benefits in healthy mother-infant dyads.

## Methods

### Trial design

This RCT included two parallel groups. The ethics committee of the Social Science faculty of the Radboud University in Nijmegen, The Netherlands (ECSW2015-2311-358) approved the study in accordance with the Declaration of Helsinki. The study was performed in accordance with this declaration. The trial was registered at the Netherlands Trial Register (NTR5697; 13/03/2016), designed and reported according to the SPIRIT and CONSORT guidelines (see Supplementary Table S7 online), published^42^, and remained unchanged after trial commencement, except for additional analyses. Since the trial did not involve any harmful procedures or adverse advents, no data-monitoring committee was involved.

### Participants

Pregnant women were recruited in Nijmegen, The Netherlands, and surrounding areas. Maternal eligibility criteria were: age ≥ 18, singleton pregnancy, no drug/medication use, no severe health problems, Dutch language proficiency, and no concurrent participation in other studies. Infant eligibility criteria were: born ≥ 37 weeks, no congenital anomalies, birthweight ≥ 2500 g, and 5-min Apgar score ≥ 7.

### Procedure

#### Prenatal phase

Pregnant women, recruited through a participant database, flyers, and social media, learned that the study was about infant sleep and feeding, mother-infant contact, mother-infant (mental) health, and that some women would be asked to implement a five-week daily mother-infant contact period. Interested, eligible women received detailed study information at a home visit between weeks 34–36 of gestation. After, written informed consent was obtained from all participants. After obtaining consent, the principal investigator opened a sealed, opaque condition assignment envelope (prepared by an independent researcher). Participants were randomly assigned (1:1) with random blocks (four and six) stratified by parity (primiparae versus multiparae) by a computer-generated random allocation sequence. SSC mothers and the principal investigator, who also analyzed the data, were not masked for group allocation. SSC mothers were encouraged to engage in at least one uninterrupted daily hour of SSC for the first five postnatal weeks (Dutch mothers are entitled to 10 to 12 weeks of paid leave after birth), starting immediately after birth (adapted from a prior study^26^). Detailed written and oral instructions on the SSC protocol, optimal SSC position and safety precautions were provided. CAU mothers received no additional instructions. All mothers were encouraged to contact the principal investigator when experiencing any problems during the study by phone, text message, or email. Besides SSC, both conditions underwent the same procedures. All mothers completed demographic and baseline questionnaires on paper.

#### Postnatal phase

During the intervention period, all mothers reported daily information on SSC, holding (clothed physical contact), and no-contact in 15-min intervals every 2–3 h in a logbook. At 2, 5, and 12-weeks postnatally, mothers completed paper and digital (only week 12) questionnaires on maternal mental health and postpartum healing except for touch discomfort (only at week 5). Researchers contacted all mothers weekly by telephone (postnatal day 5 and 13) or text-message/email (postnatal day 21 and 28) to remind mothers to complete the logbook, to ask for possible questions/comments and, for SSC mothers, to discuss SSC obstacles.

#### Follow-up

One year after birth, mothers completed digital questionnaires and had a debriefing home visit.

### Measures

#### Depressive symptoms (primary outcome)

The Edinburgh Postnatal Depression Scale (EPDS)^61^ consists of 10 items on a 4-point scale (0–3). Items were reverse scored where appropriate and summed. Higher scores indicate higher levels of depression (range = 0–30; Cronbach’s α = 0.82–0.87). A threshold of 10 indicates clinical levels of depression^62^. The Dutch translation has demonstrated good psychometric properties during the prenatal and postnatal periods (Cronbach’s α = 0.82; concurrent validity *r* range = 0.57–0.69; test–retest validity *r* range = 0.55–0.63^63,64^).

#### Anxiety symptoms

The State subscale of the State-Trait Anxiety Inventory (STAI)^65^ consists of 20 items on a 4-point scale (1–4;). Items were reversed scored where appropriate and summed. Higher scores indicate higher levels of anxiety (range = 20–80; Cronbach’s α = 0.80–0.94). A threshold of 40 indicates clinical levels of anxiety^65^. The Dutch translation has good psychometric properties in a community sample (Cronbach’s α = 0.83; concurrent validity *r* range = 0.89^66^).

#### Stress symptoms

The Everyday Problems List (EPL)^67^ consists of 49 daily hassles. Participants indicated if they experienced each hassle and how much it bothered them on a 4-point scale (1–4). Scores on how much each hassle bothered them were summed; higher scores indicate more stress symptoms (range = 0–196).

#### Fatigue symptoms

The Multidimensional Fatigue Inventory (MFI)^68^ consists of 20 items on a 5-point scale (1 – 5). Items were reverse scored where appropriate and summed. Total scores range from 20 to 100; higher scores indicate more fatigue symptoms (Cronbach’s α = 0.92–0.95 for all measurements).

#### Pain symptoms

An adjusted version of the 2-item Bodily Pain subscale of the SF-36^69^ assessed pain symptoms. Items, which were on a 6-point scale (1–6), were averaged. Higher scores indicate more pain symptoms (Cronbach’s α = 0.78–1.00 for all measurements). The Dutch translation has good psychometric properties in a community sample (Cronbach’s α = 0.84; item discriminant validity range = 0.28–0.60^70^).

#### Delivery-related post-traumatic stress symptoms

Twenty-one items of the Traumatic Event Scale–Delivery (TES-B)^71^, a 24-item questionnaire developed to diagnose delivery-related PTSD, assessed maternal PTSS. Four items assessed feelings of the birth as traumatic and 17 items assessed trauma symptoms; both were on a 4-point scale (0–3). One item on symptom severity and two items on symptom duration were excluded as they were on a different scale (e.g., the number of months experiencing symptoms). Means were calculated; higher scores indicate more PTSS (Cronbach’s α = 0.86–0.90 for all measurements).

#### Prenatal symptoms severity (potential moderator)

The EPDS (Cronbach’s α = 0.82), the State subscale of the STAI (Cronbach’s α = 0.88), and the EPL reported in late pregnancy were used to assess the severity of prenatal symptoms.

#### Touch discomfort (potential moderator)

The Social Touch Questionnaire (STQ)^72^ consists of 20 items on a 5-point scale (0–4). Item scores were summed. Higher scores indicate higher levels of touch discomfort (range = 0–80; Cronbach’s α = 0.82). No psychometric information is available on the Dutch translation.

### Statistical analyses

Sample size calculations were based on the primary outcome: maternal depressive symptoms. With 80% power, 0.05 significance level, and an effect size of Cohens *f* = 0.24, derived from a prior SSC study^26^, 58 dyads in each group were needed accounting for attrition^42^.

Outliers were winsorized by replacing values with three times the standard deviation above or below the mean^46^. Missingness was investigated. Conditions (0 = CAU; 1 = SSC) were compared on total SSC duration with Mann Whitney U tests and SS dyads that reported ≥ 28 out of 35 days with ≥ 60 min of SSC were defined.

Primary analyses consisted of 1) Intention-To-Treat (ITT), 2) Per-Protocol (PP), and 3) exploratory Dose–Response (DR) analyses. In ITT analyses, groups were compared regardless of compliance or withdrawal. For PP analyses, SSC dyads who adhered to the SSC protocol and had no missing outcome data were compared with CAU dyads without missing outcome data. SSC protocol adherence was derived from the daily contact logbook. SSC minutes were summed per day when data was registered for ≥ 80% of each day for ≥ 21 out of 35 days. Missing days were replaced with the SSC mean of two days before and two days after the missing day (based on a prior study^73^). Data from remaining logbooks was not used. Of the 116 participants, 90 adequately collected SSC data. Of these, 27 had no missing days, 39 had 1 or 2 missing days, 16 had 3 to 7 missing days, and 8 had 8 to 14 missing days. SSC dyads with ≥ 28 out of 35 days with ≥ 60 min of SSC and no missing outcome data were included in PP analyses. For exploratory DR analyses, conducted in the SSC group, the sum of SSC minutes during the intervention period was used as the independent variable.

Multilevel growth curve analyses were conducted using SPSS version 25 in Windows and version 26 in Mac OS. Time was introduced at level 1, and nested within dyad at level 2. Linear time (slope) and the intercept were random factors. First, Interclass Correlation Coefficients (ICC) were calculated, by using the null model, to examine whether the nested structure was appropriate (ICC > 0.05;^47^) for multilevel analyses. Using a build-up strategy, linear time was first entered as a fixed main effect. Next, condition/duration, prenatal symptom severity (only for depressive, anxiety, and stress symptoms), and touch discomfort were added incrementally one by one as fixed main effects. Then, the interaction of time by condition/duration, condition/duration by prenatal symptom severity (only for depressive, anxiety, and stress symptoms), and condition/duration by touch discomfort were added one by one. Last, 3-way interactions of time by condition/duration by prenatal symptom severity (only for depressive, anxiety, and stress symptoms) and time by condition/duration by touch discomfort were entered. After every inclusion, the degrees of freedom and the deviance on the -2 log likelihood ratio scale was compared to the values on the previous model. Significance was checked with the critical values for the chi-square distribution. To preserve parsimony, only terms that added significantly to the model were included in subsequent models. Normality of the residuals of the final model was checked. The best fitting models are presented in the results. The original analysis plan only specified ITT analyses; PP and exploratory DR analyses were added due to current recommendations^74^.

Hierarchical regression analyses were used to exploratorily examine 52-week follow-up effects in the ITT, PP, and DR frameworks. Condition/duration was added in the first block, prenatal symptoms severity (only for depressive, anxiety, and stress symptoms) and touch discomfort in the second, and their interactions in the third.

## Supplementary Information

Supplementary Tables.

## Data Availability

The data are not freely available, as we did not ask participants to consent to store de-identified data in an online depository. Deidentified individual participant data that underlie the results reported in this article will be made available upon publication (no end date) to researchers with a methodologically sound proposal for re-use of the data. The proposal should be directed to carolina.deweerth@radboudumc.nl. Upon approval, data requestors will need to sign a data transfer agreement. Researchers are asked to analyze the data and/or publish the results within two years.
