# Supplementary material for: Daily mother-infant skin-to-skin contact and maternal mental health and postpartum healing: a randomized controlled trial
Source: Sci Rep. 2022 Jun 17;12:10225. doi: 10.1038/s41598-022-14148-3 (PMC9205929; doi:10.1038/s41598-022-14148-3)
Supplement: Supplementary file 1 — Supplementary Tables. [file 41598_2022_14148_MOESM1_ESM.docx]

**Supplementary Information**

Article title: Daily Mother-Infant Skin-to-Skin Contact and Maternal Mental Health and Postpartum Healing: A Randomized Controlled Trial

Journal: Scientific Reports

Authors: Kelly H.M. Cooijmans, Roseriet Beijers, Bonnie E. Brett, Carolina de Weerth

Corresponding author: kelly.cooijmans@radboudumc.nl

**Supplementary Table S1.** Percentage of Missing Data, Number of Outliers, and the Winsorized Value for Outliers for all Outcome and Moderator Variables in the Total Sample

| **Supplementary table S1.** *Percentage of Missing Data, Number of Outliers, and the Winsorized Value for Outliers for all Outcome and Moderator Variables in the Total Sample* | | | | | | |
| --- | --- | --- | --- | --- | --- | --- |
|  | | *% of missingness* |  | *Number of outliers* |  | *Winsorized value* |
| *Maternal Prenatal week 34-6* | |  |  |  |  |  |
| Depressive symptoms | 0.0 |  | 2 |  | 16.34 |  |
| Anxiety symptoms | 0.9 |  | 1 |  | 51.25 |  |
| Stress symptoms | 0.9 |  | 1 |  | 39.68 |  |
| *Maternal Postnatal week 2* | |  |  |  |  |  |
| Depressive symptoms | 10.3 |  | 2 |  | 16.96 |  |
| Anxiety symptoms | 10.3 |  | 1 |  | 50.14 |  |
| Stress symptoms | 10.3 |  | 2 |  | 42.86 |  |
| Fatigue | 11.9 |  | 0 |  | -- |  |
| Pain | 11.0 |  | 0 |  | -- |  |
| Delivery-related PTSS | 11.0 |  | 2 |  | 2.27 |  |
| *Maternal Postnatal week 5* | |  |  |  |  |  |
| Depressive symptoms | 9.5 |  | 1 |  | 15.40 |  |
| Anxiety symptoms | 9.5 |  | 2 |  | 54.21 |  |
| Stress symptoms | 9.5 |  | 2 |  | 38.10 |  |
| Fatigue | 12.8 |  | 0 |  | -- |  |
| Pain | 12.8 |  | 1 |  | 5.99 |  |
| Delivery-related PTSS | 12.8 |  | 1 |  | 2.31 |  |
| *Maternal Postnatal week 12* | |  |  |  |  |  |
| Depressive symptoms | 9.5 |  | 1 |  | 18.51 |  |
| Anxiety symptoms | 10.3 |  | 1 |  | 56.04 |  |
| Stress symptoms | 11.2 |  | 2 |  | 42.37 |  |
| Fatigue | 11.0 |  | 0 |  | -- |  |
| Pain | 11.0 |  | 1 |  | 5.53 |  |
| Delivery-related PTSS | 10.1 |  | 3 |  | 2.16/ 2.26^a^ |  |
| *Maternal Postnatal week 52* | |  |  |  |  |  |
| Depressive symptoms | 10.3 |  | 2 |  | 17.65 |  |
| Anxiety symptoms | 11.2 |  | 2 |  | 56.76 |  |
| Stress symptoms | 11.2 |  | 1 |  | 51.28 |  |
| Fatigue | 10.1 |  | 0 |  | -- |  |
| Pain | 10.1 |  | 0 |  | -- |  |
| Delivery-related PTSS | 10.1 |  | 2 |  | 2.29/2.37^a^ |  |
| *Touch discomfort* | | 12.1 |  | 1 |  | 55.76 |
| *CAU*, care-as-usual; *PTSS*, post-traumatic stress symptoms; *SSC*, skin-to-skin contact.  ^a^ Outliers were winsorized by treatment group: SSC/CAU. | | | | | | |

**Supplementary Table S2.** Ranges and Median Values for Post-Traumatic Stress Symptom Scores for Postnatal Week 2, 5, 12, and 52

| **Supplementary table S2.** *Ranges and Median Values for Post-Traumatic Stress Symptom Scores for Postnatal Week 2, 5, 12, and 52* | | | | | |
| --- | --- | --- | --- | --- | --- |
|  | Intention-to-treat | |  | Per-Protocol | |
|  | SSC | CAU |  | SSC | CAU |
| Week 2 | 1.00 – 1.81 (1.24) | 1.99 – 2.27 (1.19) |  | 1.05 – 1.81 (1.24) | 1.00 – 2.27 (1.19) |
| Week 5 | 1.00 – 1.86 (1.14) | 1.00 – 2.31 (1.14) |  | 1.00 – 1.86 (1.19) | 1.00 – 2.31 (1.14) |
| Week 12 | 1.00 – 2.16 (1.14) | 1.00 – 2.26 (1.19) |  | 1.00 – 1.48 (1.14) | 1.00 – 2.26 (1.19) |
| Week 52 | 1.00 – 1.81 (1.19) | 1.00 – 2.37 (1.19) |  | 1.00 – 1.71 (1.24) | 1.00 – 2.37 (1.19) |
| *Note. CAU*, care-as-usual; *SSC*, skin-to-skin contact. All values are: Range (Median value) | | | | | |

**Supplementary Table S3.** Fit Indices Throughout the Model Building Process for Maternal Depressive, Anxiety, and Stress Symptoms Models in Intention-to-treat, Per-Protocol, Exploratory Dose-Response Analyses.

| **Supplementary table S3** *Fit Indices for the Model Building Process of Maternal Depressive, Anxiety, and Stress* *Symptoms Models in Intention-to-treat, Per-Protocol, Exploratory Dose-Response Analyses.* | | | | | | | | | | | | |
| --- | --- | --- | --- | --- | --- | --- | --- | --- | --- | --- | --- | --- |
|  | | *Intention-to-treat* | | |  | *Per-Protocol* | | |  | *Exploratory dose-response* | | |
| **Depressive** | | *-2 log likelihood* | *df* | *Chi-square difference* |  | *-2 log likelihood* | *df* | *Chi-square difference* |  | *-2 log likelihood* | *df* | *Chi-square difference* |
| Intercept (I) | 1632.76 | 0 |  |  | 1019.50 | 0 |  |  | 739.38 | 0 |  |  |
| I + Time (Ti) | 1626.23 | 1 | 6.53 |  | 1015.37 | 1 | 4.13 |  | 735.57 | 1 | 3.81 |  |
| I, Ti, Condition (C) | 1625.54 | 1 | 0.69 |  | 1014.93 | 1 | 0.44 |  | 735.50 | 1 | 3.88 |  |
| I, Ti, C, Touch Discomfort (TD) | 1550.61 | 2 | 74.93 |  | 992.49^a^ | 2 | 22.44 |  | 716.68 | 3 | 18.82 |  |
| I, Ti, C, TD, Prenatal Symptoms (PS) | 1512.05 | 1 | 38.56 |  | 977.38 | 1 | 15.11 |  | 696.03 | 1 | 20.65 |  |
| I, Ti, C, TD, PS, TD*C | 1512.04 | 1 | 0.01 |  | 977.02 | 1 | 0.36 |  | 695.91 | 1 | 0.12 |  |
| I, Ti, C, TD, PS, TD*C, C*TD | 1509.29 | 2 | 2.76 |  | 977.38^a^ | 2 | 0.00 |  | 695.99 | 2 | 0.08 |  |
| I, Ti, C, TD, PS, TD*C, C*PS | 1511.87 | 2 | 0.17 |  | 976.24 | 2 | 1.14 |  | 694.42 | 2 | 1.61 |  |
| I, Ti, C, TD, PS, TD*C, Ti*C*TD | 1510.06 | 2 | 1.98 |  | 977.33^a^ | 2 | 0.05 |  | 695.99 | 2 | 0.04 |  |
| I, Ti, C, TD, PS, TD*C, Ti*C*PS | 1515.73 | 2 | 3.69 |  | 977.22 | 2 | 0.16 |  | 697.38 | 2 | 1.35 |  |
| **Anxiety** | |  |  |  |  |  |  |  |  |  |  |  |
| Intercept (I) | 2015.23 | 0 |  |  | 1269.41 | 0 |  |  | 912.76 | 0 |  |  |
| I + Time (Ti) | 2003.63 | 1 | 11.60 |  | 1265.75 | 1 | 3.66 |  | 902.33 | 1 | 10.43 |  |
| I, Ti, Condition (C) | 2003.58 | 1 | 0.05 |  | 1265.43 | 1 | 0.33 |  | 902.23 | 1 | 0.10 |  |
| I, Ti, C, Touch Discomfort (TD) | 1921.99 | 2 | 81.59 |  | 1224.68^a^ | 2 | 40.75 |  | 865.04^a^ | 2 | 37.19 |  |
| I, Ti, C, TD, Prenatal Symptoms (PS) | 1874.53 | 1 | 47.46 |  | 1203.90 | 1 | 20.78 |  | 843.84 | 1 | 21.2 |  |
| I, Ti, C, TD, PS, TD*C | 1870.43 | 1 | 4.10 |  | 1197.18 | 1 | 6.72 |  | 843.60 | 1 | 0.24 |  |
| I, Ti, C, TD, PS, TD*C, C*TD | 1869.98 | 1 | 0.45 |  | 1197.10^a^ | 1 | 0.08 |  | 842.36^a^ | 2 | 1.48 |  |
| I, Ti, C, TD, PS, TD*C, C*PS | 1870.32 | 2 | 0.11 |  | 1196.99 | 2 | 0.11 |  | 843.55 | 2 | 0.29 |  |
| I, Ti, C, TD, PS, TD*C, Ti*C*TD | 1863.37 | 2 | 7.06 |  | 1195.09^a^ | 2 | 2.09 |  | 843.10^a^ | 2 | 0.74 |  |
| I, Ti, C, TD, PS, TD*C, Ti*C*PS | 1865.77 | 2 | 4.66 |  | 1196.17 | 2 | 1.01 |  | 843.30 | 2 | 0.54 |  |
| **Stress** | |  |  |  |  |  |  |  |  |  |  |  |
| Intercept (I) | 2073.09 | 0 |  |  | 1301.47 | 0 |  |  | 959.94 | 0 |  |  |
| I + Time (Ti) | 2062.06 | 1 | 11.03 |  | 1288.22 | 1 | 13.25 |  | 956.30 | 1 | 3.64 |  |
| I, Ti, Condition (C) | 2062.06 | 1 | 0.00 |  | 1288.22 | 1 | 0.00 |  | 956.13 | 1 | 0.17 |  |
| I, Ti, C, Touch Discomfort (TD) | 1971.38 | 2 | 90.68 |  | 1261.07^a^ | 2 | 27.15 |  | 906.72^a^ | 3 | 49.41 |  |
| I, Ti, C, TD, Prenatal Symptoms (PS) | 1908.95 | 1 | 62.43 |  | 1239.41 | 1 | 21.66 |  | 889.71 | 1 | 17.01 |  |
| I, Ti, C, TD, PS, TD*C | 1908.87 | 1 | 0.08 |  | 1237.70 | 1 | 1.71 |  | 887.95 | 1 | 1.76 |  |
| I, Ti, C, TD, PS, TD*C, C*TD | 1908.64 | 2 | 0.31 |  | 1239.09^a^ | 2 | 0.32 |  | 887.94^a^ | 2 | 1.77 |  |
| I, Ti, C, TD, PS, TD*C, C*PS | 1908.38 | 2 | 0.57 |  | 1239.39 | 2 | 0.02 |  | 885.06 | 2 | 4.65 |  |
| I, Ti, C, TD, PS, TD*C, Ti*C*TD | 1908.39 | 2 | 0.56 |  | 1235.95^a^ | 2 | 3.46 |  | 887.98^a^ | 2 | 1.73 |  |
| I, Ti, C, TD, PS, TD*C, Ti*C*PS | 1908.18 | 2 | 0.77 |  | 1237.98 | 2 | 1.46 |  | 883.57 | 2 | 6.14 |  |
| *Note.*   *C*, condition; *I,* intercept; *PS*, prenatal mental health symptoms; *TD*; touch discomfort; *Ti,* time. The grey shadowing represents the best fitting model.  ^a^ For the best fitting final multilevel models, prenatal mental health symptoms were included before touch discomfort. | | | | | | | | | | | | |

**Supplementary Table S4.** Fit Indices Throughout the Model Building Process for Maternal Fatigue and Pain Symptoms Models in Intention-to-treat, Per-Protocol, Exploratory Dose-Response Analyses.

| **Supplementary table S4.** *Fit Indices Throughout the Model Building Process for Maternal Fatigue and Pain Symptoms Models in Intention-to-treat, Per-Protocol, Exploratory Dose-Response Analyses* | | | | | | | | | | | | |
| --- | --- | --- | --- | --- | --- | --- | --- | --- | --- | --- | --- | --- |
|  | | *Intention-to-treat* | | |  | *Per-Protocol* | | |  | *Exploratory dose-response* | | |
| **Fatigue** | | *-2 log likelihood* | *df* | *Chi-square difference* |  | *-2 log likelihood* | *df* | *Chi-square difference* |  | *-2 log likelihood* | *df* | *Chi-square difference* |
| Intercept (I) | 2270.03 | 0 |  |  | 1440.04 | 0 |  |  | 1949.88 | 0 |  |  |
| I + Time (Ti) | 2252.32 | 1 | 17.71 |  | 1432.63 | 1 | 7.408 |  | 1927.65 | 1 | 22.23 |  |
| I, Ti, Condition (C) | 2248.26 | 1 | 4.06 |  | 1430.19 | 1 | 2.442 |  | 1927.04 | 1 | 0.51 |  |
| I, Ti, C, Touch Discomfort (TD) | 2182.90 | 1 | 65.35 |  | 1428.04 | 1 | 2.15 |  | 1900.90 | 2 | 26.14 |  |
| I, Ti, C, TD, Ti*C | 2171.75 | 1 | 11.15 |  | 1426.11 | 1 | 1.93 |  | 1898.99 | 1 | 1.91 |  |
| I, Ti, C, TD, Ti*C, TD*C | 2171.59 | 1 | 0.16 |  | 1425.51 | 1 | 0.60 |  | 1898.83 | 1 | 0.16 |  |
| I, Ti, C, TD, Ti*C, TD*C, T*C*TD | 2168.26 | 1 | 3.33 |  | 1425.47 | 1 | 0.04 |  | 1895.59 | 1 | 3.24 |  |
|  |  |  |  |  |  |  |  |  |  |  |  |  |
| **Pain** | |  |  |  |  |  |  |  |  |  |  |  |
| Intercept (I) | 969.91 |  |  |  | 616.89 |  |  |  | 835.69 |  |  |  |
| I + Time (Ti) | 865.06 | 1 | 104.85 |  | 556.94 | 1 | 59.95 |  | 750.01 | 1 | 85.68 |  |
| I, Ti, Condition (C) | 862.44 | 1 | 2.62 |  | 555.35 | 1 | 1.59 |  | 749.71 | 1 | 0.30 |  |
| I, Ti, C, Touch (TD) | 834.90 | 2 | 27.54 |  | 555.35 | 1 | 0.02 |  | 740.51 | 2 | 9.20 |  |
| I, Ti, C, TD, Ti*C | 834.45 | 1 | 0.45 |  | 554.22 | 1 | 1.00 |  | 740.47 | 1 | 0.04 |  |
| I, Ti, C, TD, Ti*C, TD*C | 832.53 | 1 | 1.92 |  | 554.21 | 1 | 0.02 |  | 740.34 | 1 | 0.13 |  |
| I, Ti, C, TD, Ti*C, TD*C, Ti*C*TD | 828.30 | 1 | 4.23 |  | 550.07 | 1 | 4.15 |  | 737.35 | 1 | 2.99 |  |
| *Note.*   *C,* condition;  *I,* intercept; *TD*; touch discomfort; *Ti,* time. The grey shadowing represents the best fitting model. | | | | | | | | | | | | |

w

**Supplementary Table S5.** Results from Hierarchical Regression Analyses for Maternal Depressive, Anxiety, and Stress Symptoms During the Follow-Up 52 Weeks Postnatally

| **Supplementary table S5**.  *Results from Hierarchical* *Regression Analyses for Maternal Depressive, Anxiety, and Stress symptoms During the Follow-Up 52 Weeks Postnatally* | | | | | | | | | | | | | | | | | | |
| --- | --- | --- | --- | --- | --- | --- | --- | --- | --- | --- | --- | --- | --- | --- | --- | --- | --- | --- |
|  | | Intention-To-Treat | | |  |  |  | Per-Protocol | | |  |  |  | Dose-Response | | |  |  |
|  | | *B* (*SE*) | *p* | 95% CI | *R^2^*_model_ | *F* _change_*(p)* |  | *B* (*SE*) | *p* | 95% CI | *R^2^*_model_ | *F* _change_*(p)* |  | *B* (*SE*) | *p* | 95% CI | *R^2^*_model_ | *F* _change_*(p)* |
| **Depressive symptoms** | |  |  |  |  |  |  |  |  |  |  |  |  |  |  |  |  |  |
| *Step 1* | |  |  |  |  |  |  |  |  |  |  |  |  |  |  |  |  |  |
| Condition | -0.13 (0.16) | .439 | [-0.45, 0.20] | 0.01 | 0.60(0.439) |  | -0.02(0.22) | 0.917 | [-0.46, 0.41] | 0.01 | 0.01(0.917) |  | 0.00(0.00)^a^ | 0.045 | [0.00, 0.01] | 0.09 | 4.28(0.045) |  |
| *Step 2* | |  |  |  | 0 |  |  |  |  |  | 0 |  |  |  |  |  |  |  |
| Condition | -0.18(0.14) | .199 | [-0.46, 0.10] | 0.27 | 16.84(0.001) |  | -0.01(0.20) | 0.979 | [0.40, 0.39] | 0.20 | 7.72(0.001) |  | 0.00(0.00)^a^ | 0.075 | [0.00, 0.01] | 0.28 | 5.42(0.008) |  |
| Prenatal symptoms | 0.53(0.10) | .001 | [0.34, 0.72] |  |  |  | 0.49(0.13) | 0.001 | [0.24, 0.74] |  |  |  | 0.46(0.14) | 0.002 | [0.18, 0.74] |  |  |  |
| Touch discomfort | 0.01(0.01) | .458 | [-0.01, 0.02] |  |  |  | -0.01(0.01) | 0.810 | [-0.02, 0.02] |  |  |  | -0.01(0.01) | 0.738 | [-0.03, 0.02] |  |  |  |
| *Step 3* | |  |  |  |  |  |  |  | 0 |  |  |  |  |  |  |  |  |  |
| Condition | -0.18(0.14) | .205 | [-0.47, 0.10] | 0.27 | 0.19(0.830) |  | -0.02 (0.20) | 0.910 | [-0.38, 0.42] | 0.23 | 1.56(0.217) |  | 0.00(0.00)^a^ | 0.103 | [0.00, 0.00] | 0.35 | 1.93(0.159) |  |
| Prenatal symptoms | 0.48(0.14) | .001 | [0.21, 0.75] |  |  |  | 0.48(0.14) | 0.001 | [0.20, 0.75] |  |  |  | 0.48(0.15) | 0.002 | [0.18, 0.77] |  |  |  |
| Touch discomfort | 0.01(0.01) | .480 | [-0.01, 0.03] |  |  |  | 0.01(0.01) | 0.487 | [-0.02, 0.03] |  |  |  | 0.01(0.01) | 0.771 | [-0.02, 0.03] |  |  |  |
| Condition by PS | 0.11(0.19) | .564 | [-0.04, 0.03] |  |  |  | 0.07(0.31) | 0.822 | [-0.55, 0.69] |  |  |  | 0.00(0.00) ^a^ | 0.650 | [-0.01, 0.00] |  |  |  |
| Condition by TD | -0.01(0.02) | .781 | [-0.27, 0.49] |  |  |  | -0.04(0.02) | 0.082 | [-0.08, 0.01] |  |  |  | 0.00(0.00)^a^ | 0.092 | [0.00, 0.00] |  |  |  |
| **Anxiety symptoms** | |  |  |  |  |  |  |  |  |  |  |  |  |  |  |  |  |  |
| *Step 1* | |  |  |  |  |  |  |  | 0 |  |  |  |  |  |  |  |  |  |
| Condition | -0.01(0.01) | .577 | [-0.01, 0.01] | 0.01 | 0.31(0.577) |  | 0.00(0.01) | 0.817 | [-0.01, 0.01] | 0.01 | 0.05 (0.817) |  | 0.00(0.00)^a^ | 0.126 | [0.00, 0.00] | 0.06 | 2.43 (0.126) |  |
| *Step 2* | |  |  |  |  |  |  |  | 0 |  |  |  |  |  |  |  |  |  |
| Condition | -0.01(0.01) | .404 | [-0.01, 0.01] | 0.28 | 18.05(0.001) |  | 0.00(0.01) | 0.927 | [-0.01, 0.01] | 0.15 | 5.40(0.007) |  | 0.00(0.00)^a^ | 0.371 | [0.00, 0.00] | 0.34 | 8.51(0.001) |  |
| Prenatal symptoms | 0.55(0.10) | .001 | [0.35, 0.74] |  |  |  | 0.40(0.13) | 0.002 | [0.15, 0.66] |  |  |  | 0.56(0.14) | 0.001 | [0.28, 0.84] |  |  |  |
| Touch discomfort | 0.01(0.01) | .050 | [0.01, 0.01] |  |  |  | 0.00(0.00) | 0.440 | [0.00, 0.00] |  |  |  | 0.00(0.00) | 0.329 | [0.00, 0.00] |  |  |  |
| *Step 3* | |  |  |  |  |  |  |  |  |  |  |  |  |  |  |  |  |  |
| Condition | -0.01(0.01) | .451 | [-0.01, 0.01] | 0.29 | 0.37(0.690) |  | 0.00(0.01) | 0.901 | [-0.01, 0.01] | 0.17 | 0.90(0.414) |  | 0.00(0.00)^a^ | 0.464 | [0.00, 0.00] | 0.39 | 1.80(0.179) |  |
| Prenatal symptoms | 0.44(0.16) | .006 | [0.13, 0.76] |  |  |  | 0.44(0.15) | 0.005 | [0.14, 0.75] |  |  |  | 0.57(0.14) | 0.001 | [0.29, 0.85] |  |  |  |
| Touch discomfort | 0.01 (0.01) | .203 | [0.01, 0.01] |  |  |  | 0.00(0.00) | 0.197 | [0.00, 0.00] |  |  |  | 0.00(0.00) | 0.145 | [0.00, 0.00] |  |  |  |
| Condition by PS | 0.17(0.20) | .410 | [-0.24, 0.57] |  |  |  | -0.13(0.27) | 0.643 | [-0.67, 0.42] |  |  |  | 0.00(0.00)^a^ | 0.275 | [-0.01, 0.00] |  |  |  |
| Condition by TD | 0.01(0.01) | .843 | [0.01, 0.01] |  |  |  | 0.00(0.00) | 0.215 | [-0.01, 0.00] |  |  |  | 0.00(0.00)^a^ | 0.093 | [0.00, 0.00] |  |  |  |
| **Stress symptoms** | |  |  |  |  |  |  |  |  |  |  |  |  |  |  |  |  |  |
| *Step 1* | |  |  |  |  |  |  |  |  |  |  |  |  |  |  |  |  |  |
| Condition | 0.39(2.20) | .860 | [-3.98, 4.76] | 0.00 | 0.03(0.860) |  | -1.75(2.92) | 0.551 | [-7.58, 4.08] | 0.01 | 0.36(0.551) |  | -0.01(0.01) | 0.548 | [-0.01, 0.01] | 0.01 | 0.37(0.548) |  |
| *Step 2* | |  |  |  |  |  |  |  |  |  |  |  |  |  |  |  |  |  |
| Condition | -.1.28(1.85) | .490 | [-4.96, 2.39] | 0.33 | 22.59(0.001) |  | -3.01(2.71) | 0.272 | [-8.43, 2.42] | 0.18 | 6.86(0.002) |  | -0.01(0.01) | 0.260 | [-0.01, 0.01] | 0.40 | 13.11(0.001) |  |
| Prenatal symptoms | 0.78(0.10) | .001 | [0.55, 1.02] |  |  |  | 0.61(0.17) | 0.001 | [0.28, 0.95] |  |  |  | 0.75(0.15) | 0.001 | [0.45, 1.05] |  |  |  |
| Touch discomfort | 0.12(0.10) | .252 | [-0.09, 0.33] |  |  |  | 0.04(0.13) | 0.753 | [-0.22, 0.31] |  |  |  | 0.08(0.15) | 0.614 | [-0.23, 0.38] |  |  |  |
| *Step 3* | |  |  |  |  |  |  |  |  |  |  |  |  |  |  |  |  |  |
| Condition | -1.28(1.87) | .496 | [-4.99, 2.43] | 0.33 | 0.15(0.859) |  | -2.66(2.66) | 0.323 | [-7.98, 2.67] | 0.24 | 2.39(0.100) |  | -0.01(0.01) | 0.178 | [-0.01, 0.01] | 0.47 | 2.67(0.082) |  |
| Prenatal symptoms | 0.84(0.18) | .001 | [0.47, 1.20] |  |  |  | 0.84(0.19) | 0.001 | [0.45, 1.22] |  |  |  | 0.81(0.15) | 0.001 | [0.52, 1.10] |  |  |  |
| Touch discomfort | 0.09(0.15) | .552 | [-0.20, 0.38] |  |  |  | 0.09(0.15) | 0.572 | [-0.22, 0.39] |  |  |  | 0.09(0.16) | 0.565 | [-0.22, 0.41] |  |  |  |
| Condition by PS | -0.10(0.24) | .693 | [-0.57, 0.38] |  |  |  | -0.78(0.36) | 0.035 | [-1.49, -0.06] |  |  |  | 0.00(0.00)^a^ | 0.043 | [-0.01, 0.00] |  |  |  |
| Condition by TD | 0.08(0.21) | .719 | [-0.34, 0.50] |  |  |  | -0.13(0.30) | 0.664 | [-0.72, 0.46] |  |  |  | 0.00(0.00)^a^ | 0.823 | [0.00, .00] |  |  |  |
| *PS,* Prenatal symptoms*; TD*, Touch discomfort. ^a^ Values are for dose variable rather than condition variable. | | | | | | | | | | | | | | | | | | |

**Supplementary Table S6.** Results from Hierarchical Regression Analyses for Maternal Fatigue and Pain Symptoms During the Follow-Up 52 Weeks Postnatally

| **Supplementary table S6.**  *Results from Hierarchical* *Regression Analyses for Maternal Fatigue and Pain During the Follow-Up 52 Weeks Postnatally.* | | | | | | | | | | | | | | | | | | |
| --- | --- | --- | --- | --- | --- | --- | --- | --- | --- | --- | --- | --- | --- | --- | --- | --- | --- | --- |
|  | | Intention-To-Treat | | |  |  |  | Per-Protocol | | |  |  |  | Dose-Response | | |  |  |
|  | | *B* (*SE*) | *p* | 95% CI | *R^2^*_model_ | *F* _change_*(p)* |  | *B* (*SE*) | *p* | 95% CI | *R^2^*_model_ | *F* _change_*(p)* |  | *B* (*SE*) | *p* | 95% CI | *R^2^*_model_ | *F* _change_*(p)* |
| **Fatigue** | |  |  |  |  |  |  |  |  |  |  |  |  |  |  |  |  |  |
| *Step 1* | |  |  |  |  |  |  |  |  |  |  |  |  |  |  |  |  |  |
| Condition | -3.75 (3.30) | 0.258 | [-10.29, 2.80] | 0.01 | 1.29 (0.258) |  | -2.18 (4.92) | 0.660 | [-12.03, 7.67] | 0.00 | 0.20 (0.660) |  | 0.00 (0.00)^a^ | 0.890 | [0.00, 0.00] | 0.00 | 0.02 (0.890) |  |
| *Step 2* | |  |  |  |  |  |  |  |  |  |  |  |  |  |  |  |  |  |
| Condition | -3.30 (3.26) | 0.314 | [-9.77, 3.17] | 0.05 | 3.68 (0.058) |  | -2.82 (4.90) | 0.567 | [-12.64, 6.99] | 0.04 | 1.99 (0.164) |  | 0.00 (0.00)^a^ | 0.967 | [0.00, 0.00] | 0.04 | 2.50 (0.120) |  |
| Touch discomfort | 0.38 (0.20) | 0.058 | [-0.01, 0.78] |  |  |  | 0.37 (0.26) | 0.164 | [-0.16, 0.90] |  |  |  | 0.42 (0.27) | 0.120 | [-0.11, 0.95] |  |  |  |
| *Step 3* | |  |  |  |  |  |  |  |  |  |  |  |  |  |  |  |  |  |
| Condition | -3.36 (3.28) | 0.309 | [-9.88, 3.17] | 0.05 | 0.05 (0.817) |  | -2.88 (4.99) | 0.566 | [-12.87, 7.11] | 0.04 | 0.01 (0.927) |  | 0.00 (0.00)^a^ | 0.913 | [0.00, 0.00] | 0.04 | 0.04 (0.852) |  |
| Touch discomfort | 0.38 (0.20) | 0.060 | [-0.02, 0.78] |  |  |  | 0.38 (0.27) | 0.167 | [-0.16, 0.91] |  |  |  | 0.44 (0.30) | 0.140 | [-0.15, 1.04] |  |  |  |
| Condition by TD | -0.09 (0.40) | 0.817 | [-0.90, 0.71] |  |  |  | 0.06 (0.67) | 0.927 | [-1.27, 1.40] |  |  |  | 0.00 (0.00)^a^ | 0.852 | [0.00, 0.01] |  |  |  |
| **Pain** | |  |  |  |  |  |  |  |  |  |  |  |  |  |  |  |  |  |
| *Step 1* | |  |  |  |  |  |  |  |  |  |  |  |  |  |  |  |  |  |
| Condition | 0.01 (0.21) | 0.979 | [-0.41, 0.42] | 0.00 | 0.00 (0.979) |  | 0.18 (0.30) | 0.555 | [-0.43, 0.79] | 0.01 | 0.35 (0.555) |  | 0.00 (0.00)^a^ | 0.510 | [0.00, 0.00] | 0.01 | 0.44 (0.510) |  |
| *Step 2* | |  |  |  |  |  |  |  |  |  |  |  |  |  |  |  |  |  |
| Condition | 0.02 (0.21) | 0.925 | [-0.40, 0.44] | 0.01 | 0.88 (0.350) |  | 0.18 (0.31) | 0.564 | [-0.44, 0.79] | 0.01 | 0.01 (0.947) |  | 0.00 (0.00)^a^ | 0.517 | [0.00, 0.00] | 0.01 | 0.00 (0.992) |  |
| Touch discomfort | 0.01 (0.01) | 0.350 | [-0.01, 0.04] |  |  |  | 0.01 (0.02) | 0.947 | [-0.03, 0.03] |  |  |  | 0.00 (0.02) | 0.992 | [-0.03, 0.03] |  |  |  |
| *Step 3* | |  |  |  |  |  |  |  |  |  |  |  |  |  |  |  |  |  |
| Condition | 0.02 (0.21) | 0.935 | [-0.40, 0.44] | 0.01 | 0.03 (0.868) |  | 0.22 (0.31) | 0.471 | [-0.40, 0.84] | 0.03 | 1.33 (0.253) |  | 0.00 (0.00)^a^ | 0.255 | [0.00, 0.00] | 0.04 | 2.04 (0.159) |  |
| Touch discomfort | 0.01 (0.01) | 0.354 | [-0.01, 0.04] |  |  |  | -0.01 (0.02) | 0.941 | [-0.04, 0.03] |  |  |  | -0.01 (0.02) | 0.552 | [-0.05, 0.03] |  |  |  |
| Condition by TD | 0.00 (0.03) | 0.868 | [-0.06, 0.05] |  |  |  | -0.05 (0.04) | 0.253 | [-0.13, 0.04] |  |  |  | 0.00 (0.00)^a^ | 0.159 | [0.00, 0.00] |  |  |  |
| *TD*, Touch discomfort.  ^a^ Values are for dose variable rather than condition variable. | | | | | | | | | | | | | | | | | | |

**Supplementary Table S7**. CONSORT Checklist


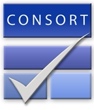
Supplementary Table S7. CONSORT 2010 checklist of information to include when reporting a randomised trial*

| Section/Topic | Item No | Checklist item | Reported on page No |
| --- | --- | --- | --- |
| Title and abstract | | | |
|  | 1a | Identification as a randomised trial in the title | 1 |
|  | 1b | Structured summary of trial design, methods, results, and conclusions (for specific guidance see CONSORT for abstracts) | 2 |
| Introduction | | | |
| Background and objectives | 2a | Scientific background and explanation of rationale | 3-5 |
|  | 2b | Specific objectives or hypotheses | 5,6 |
| Methods | | | |
| Trial design | 3a | Description of trial design (such as parallel, factorial) including allocation ratio | 16 |
|  | 3b | Important changes to methods after trial commencement (such as eligibility criteria), with reasons | 16 |
| Participants | 4a | Eligibility criteria for participants | 16 |
|  | 4b | Settings and locations where the data were collected | 16 |
| Interventions | 5 | The interventions for each group with sufficient details to allow replication, including how and when they were actually administered | 16,17 |
| Outcomes | 6a | Completely defined pre-specified primary and secondary outcome measures, including how and when they were assessed | 17-19 |
|  | 6b | Any changes to trial outcomes after the trial commenced, with reasons | 16 |
| Sample size | 7a | How sample size was determined | 19 |
|  | 7b | When applicable, explanation of any interim analyses and stopping guidelines | Not applicable |
| Randomisation: |  |  |  |
| Sequence generation | 8a | Method used to generate the random allocation sequence | 16,17 |
|  | 8b | Type of randomisation; details of any restriction (such as blocking and block size) | 16,17 |
| Allocation concealment mechanism | 9 | Mechanism used to implement the random allocation sequence (such as sequentially numbered containers), describing any steps taken to conceal the sequence until interventions were assigned | 16,17 |
| Implementation | 10 | Who generated the random allocation sequence, who enrolled participants, and who assigned participants to interventions | 16,17 |
| Blinding | 11a | If done, who was blinded after assignment to interventions (for example, participants, care providers, those assessing outcomes) and how | 16,17 |
|  | 11b | If relevant, description of the similarity of interventions | 17 |
| Statistical methods | 12a | Statistical methods used to compare groups for primary and secondary outcomes | 19-21 |
|  | 12b | Methods for additional analyses, such as subgroup analyses and adjusted analyses | 19-21 |
| Results | | | |
| Participant flow (a diagram is strongly recommended) | 13a | For each group, the numbers of participants who were randomly assigned, received intended treatment, and were analysed for the primary outcome | 6, Figure 1 |
|  | 13b | For each group, losses and exclusions after randomisation, together with reasons | 6, Figure 1 |
| Recruitment | 14a | Dates defining the periods of recruitment and follow-up | 6 |
|  | 14b | Why the trial ended or was stopped | Not applicable |
| Baseline data | 15 | A table showing baseline demographic and clinical characteristics for each group | Table 1 |
| Numbers analysed | 16 | For each group, number of participants (denominator) included in each analysis and whether the analysis was by original assigned groups | Figure 1 |
| Outcomes and estimation | 17a | For each primary and secondary outcome, results for each group, and the estimated effect size and its precision (such as 95% confidence interval) | 7-9 Table 2-4, Supplementary table S1-S6 |
|  | 17b | For binary outcomes, presentation of both absolute and relative effect sizes is recommended | Not applicable |
| Ancillary analyses | 18 | Results of any other analyses performed, including subgroup analyses and adjusted analyses, distinguishing pre-specified from exploratory | 7-9 Table 2-4, Supplementary table S1-S6 |
| Harms | 19 | All important harms or unintended effects in each group (for specific guidance see CONSORT for harms) | 6 |
| Discussion | | | |
| Limitations | 20 | Trial limitations, addressing sources of potential bias, imprecision, and, if relevant, multiplicity of analyses | 9-15 |
| Generalisability | 21 | Generalisability (external validity, applicability) of the trial findings | 9-15 |
| Interpretation | 22 | Interpretation consistent with results, balancing benefits and harms, and considering other relevant evidence | 9-15 |
| Other information | | |  |
| Registration | 23 | Registration number and name of trial registry | 14,16 |
| Protocol | 24 | Where the full trial protocol can be accessed, if available | 5,14 |
| Funding | 25 | Sources of funding and other support (such as supply of drugs), role of funders | 32 |

*We strongly recommend reading this statement in conjunction with the CONSORT 2010 Explanation and Elaboration for important clarifications on all the items. If relevant, we also recommend reading CONSORT extensions for cluster randomised trials, non-inferiority and equivalence trials, non-pharmacological treatments, herbal interventions, and pragmatic trials. Additional extensions are forthcoming: for those and for up to date references relevant to this checklist, see [www.consort-statement.org](http://www.consort-statement.org).
